# Management and Outcome of Recurring Low-Grade Intramedullary Astrocytomas

**DOI:** 10.3390/cancers16132417

**Published:** 2024-06-30

**Authors:** Elly Chaskis, Martina Silvestri, Nozar Aghakhani, Fabrice Parker, Steven Knafo

**Affiliations:** 1 Department of Neurosurgery, Bicêtre Hospital, AP-HP, 94270 Le Kremlin-Bicêtre, France; 2 Faculty of Medicine, University Paris-Saclay, 94270 Le Kremlin-Bicêtre, France

**Keywords:** spinal cord, intramedullary, astrocytoma, surgery, low grade

## Abstract

**Simple Summary:**

Low-grade intramedullary astrocytomas (LG-IMAs) are rare tumors which are most frequently considered as benign but with high recurrence rates and for which treatment after initial surgery remains unclear. In a single-center cohort including 30 patients with a median follow-up of 59 months (range = 13–376), the recurrence rate of LG-IMA was high (53.3%) and determined by the extent of surgical resection rather than histological grading. The management of recurring tumors was very variable but overall survival at 10 years remained good (81.9%).

**Abstract:**

Intramedullary astrocytomas (IMAs) are the second most frequent intramedullary tumors in adults. Low-grade IMAs (LG-IMA, WHO grade I and II) carry a better prognosis than high-grade IMAs (HG-IMAs). However, adjuvant treatment of LG-IMAs by radiotherapy (RT) and/or chemotherapy (CT) as well as treatment of tumor recurrences remains controversial. The aim of our study was to evaluate the postoperative outcome of LG-IMAs and the management of recurring tumors. We retrospectively reviewed a series of patients operated on for IMA from 1980 to 2022 in a single neurosurgical department. We retrieved 40 patients who received surgery for intramedullary astrocytomas, including 30 LG-IMAs (22 WHO grade I; 5 WHO grade II; 3 “low-grade”) and 10 HG-IMAs (4 WHO grade III; 5 WHO grade IV; 1 “high-grade”). Of the patients with LG-IMAs, the extent of surgical resection was large (gross or subtotal resection >90%) in 30% of cases. Immediate postoperative radiotherapy and/or chemotherapy was proposed only to patients who underwent biopsy (n = 5), while others were initially followed-up. Over a median follow-up of 59 months (range = 13–376), 16 LG-IMA (53.3%) recurred with a mean delay of 28.5 months after surgery (range = 3–288). These included seven biopsies, five partial resections (PR), four subtotal resections (STR) but no gross total resections (GTR). Progression-free survival for LG-IMAs was 51.9% at 3 years and 35.6% at 5 and 10 years; overall survival was 96.3% at 3 years; 90.9% at 5 years and 81.9% at 10 years. There were no significant differences in terms of OS and PFS between WHO grade I and grade II tumors. However, “large resections” (GTR or STR), as opposed to “limited resections” (PR and biopsies), were associated with both better OS (*p* = 0.14) and PFS (*p* = 0.04). The treatment of recurrences consisted of surgery alone (n = 3), surgery with RT and/or CT (n = 2), RT with CT (n = 3), RT alone (n = 2) or CT alone (n = 2). In conclusion, although LG-IMAs are infiltrating tumors, the extent of resection (GTR or STR), but not WHO grading, is the main prognostic factor. The management of recurring tumors is highly variable with no conclusive evidence for either option.

## 1. Introduction

Intramedullary astrocytomas (IMA) represent 6–8% [1,2] of spinal cord tumors. They are the second most frequent intramedullary tumor in adults after ependymoma, accounting for 30 to 40% of cases [3,4,5,6]. IMAs are classified into four grades according to the WHO classification. WHO grade I, or pilocytic, astrocytoma (IMA-I), and WHO grade II astrocytoma (IMA-II), are both low-grade tumors and the most frequently reported [7,8]. High-grade tumors include WHO grade III astrocytoma (IMA-III) and WHO grade IV glioblastoma (IM-GBM) [8].

Low-grade IMAs (LG-IMA) carry a better prognosis with a 5-year overall survival (5-OS) rate of 82% in grade I and 70% in grade II [8]. High-grade tumors have a worse prognosis with 5-OS of 28% in grade III and 14% in grade IV [4]. Tumor grade [5,9,10,11,12,13,14], extent of resection [1,4,7,9,11,15,16], preoperative neurological status [2,5,9,15,17] and adjuvant therapy [10] are favorable prognostic factors for overall survival in IMAs. Tumor recurrence in LG-IMAs is high, ranging from 21.2% [11] to 41.2% [18].

Surgery is the treatment of choice in patients with symptomatic tumors [11], aimed at a maximal resection with preservation of neurological function. Surgery must be performed as early as symptoms appear as surgical results are better when neurological signs are slight [11]. The extent of resection in IMA is often limited, as compared to ependymoma, because of its infiltrating character [10], making gross total resection (GTR) rarely achievable. Grade II, III and IV IMAs are also called “infiltrating” IMAs [10] while grade I IMAs are usually considered well-circumscribed tumors [19] offering a good dissection plane that more frequently allows a complete resection to be achieved, with a GTR rate ranging from 50 to 81% [3,20]. In some studies, however, an infiltrating behavior has been reported in IMA-1 [21].

The postoperative treatment of IMAs remains controversial. Reported data on surgery and adjuvant radiotherapy (RT) or chemotherapy (CT), by temozolomide and/or bevacizumab [17], are conflicting [9,12,22,23]. In LG-IMA, extensive surgery remains the gold standard of treatment with adjuvant RT and/or CT suggested in cases of residual tumor or recurrence [22,23]. In HG-IMAs, the beneficial effect of extensive resection is debated [13] and adjuvant RT and/or CT is recommended even if their benefits remain unclear. Nevertheless, some authors failed to demonstrate increased PFS or OS after irradiation or CT for LG-IMA or HG-IMA [1,5,6,22] and reported even shorter PFS and OS after RT [6,12] and CT [12].

The aim of our study was to evaluate long-term outcomes of LG-IMAs, and particularly the management of recurring tumors.

## 2. Materials and Methods

We reviewed all patients operated on for an IMA in the Department of Neurosurgery of Bicêtre Hospital (University Paris-Saclay) from 1980 to 2022. We retrospectively collected all preoperative, operative, and postoperative clinical and imaging data from medical records, surgical reports, and follow-up magnetic resonance imaging (MRI). We excluded patients aged under 16 and patients with incomplete medical data. 

The extent of resection was classified according to both the surgical report and the early postoperative MRI. Surgery was classified as: gross total resection (GTR) when complete resection was reported by the surgeon and no residual tumor was visible on MRI; subtotal resection (STR) when a small residual tumor was left over according to the surgical report or measured as being less than 10% of the initial tumor volume on MRI; partial resection (PR) when the remnant was larger than 10% of the initial tumor volume on MRI. 

The clinical status was evaluated according to the modified McCormick scale [24]. Tumor growth on MRI during follow-up was classified as tumor progression, even in the absence of new neurological symptoms related to this tumoral progression. Statistical analysis was performed using IBM^®^ SPSS^®^ Statistics, 1.0.-7955 22.0, using Kaplan–Meier for survival analysis.

## 3. Results

### 3.1. Patients

#### 3.1.1. Screening

We screened 48 patients with complete medical records operated on for an IMA in our department from January 1980 to December 2022. Eight patients were excluded: five patients were aged 3 to 10 years, two patients had an unclear anatomopathological diagnosis, and one patient had an incomplete medical record. 

Among these 40 IMAs, 22 cases were classified as grade I (pilocytic, IMA-I), 5 as grade II (diffuse, IMA-II), 4 as grade III (anaplastic) and 5 as grade IV (glioblastomas). In three cases, the tumor was classified as “low-grade IMA” and in one patient as “high-grade IMA”, without further grading. Altogether, we included 30 patients with LG-IMA in the present study. 

#### 3.1.2. Baseline Features

There were 18 female patients (60%); mean age was 28.5 (range = 16–66) years old. The onset of symptoms before surgery was progressive in all patients with a mean delay of 18 months (range = 1–120) before diagnosis. Sensory deficit was the most frequent presenting symptom, reported in 90% of the patients with LG-IMA, followed by pain (83.3%), motor weakness (76.7%) and sphincter disturbances (43.3%). 

Most patients with LG-IMAs (80%), including 90.9% of IMA-I, were able to walk independently before surgery (McCormick grade I and II), with only 6/30 patients requiring assistance (McCormick grade III and IV). In contrast, most patients with HG-IMA (60%) needed assistance before surgery (McCormick grade III, IV and V), 40% of the patients being McCormick grade II.

Tumor extension on imaging covered three vertebral levels on average (range: 1–9). The majority of LG-IMA (86.7%) were enhanced on gadolinium T1-weighted sequences and were associated with an intramedullary cyst in 62% of the cases. One IMA-I presented with diffuse leptomeningeal enhancement (Table 1).

### 3.2. Surgery

#### 3.2.1. Extent of Resection

Most surgeries (28/30) were performed without intraoperative electrophysiological monitoring. According to the surgical reports, only four patients (13.3% of LG-IMA cases, including two IMA-I, one IMA-II and one “low-grade”) had a delimited pseudo-dissection plan between the tumor and the spinal cord parenchyma. GTR was reported in two cases and resection was large (GTR or STR, i.e., less than 10% of residual tumor on postoperative MRI) in 10 out of 30 patients (33.3% of cases: 6 IMA-I; 2 IMA-II and 2 unspecified LG-IMA). Partial resection (>10% remnant) was obtained in 7 out of 30 patients (23.3%) while a biopsy only was performed in 13 out of 30 patients (43.3%) (Table 1). 

#### 3.2.2. Anatomopathological Results

Pathological examination according to the WHO classification in our series concluded with 22 IMA-I; 5 IMA-II and 3 LG-IMA of undefined grade. Ki67 level was between 1 and 10 in LG-IMA (<5% in 90.9% of cases) (Table 2). No IDH-mutation was found in any of the 13 patients tested. A BRAF-mutation was detected in four of six tested IMA-I (66.7%) and absent in one IMA-II. H3K27m mutation was present in the only tested IMA-II but absent in the three tested IMA-I.

#### 3.2.3. Early Postoperative Outcome

Clinical worsening (1 or 2 grades on McCormick scale) on immediate postoperative examination was observed in 30% of patients. Clinical improvement was noted in one patient with IMA-I (McCormick grade II improving to a grade I after surgery). The two patients with GTR presented a postoperative neurological worsening (grade III to V and grade II to III, respectively). One patient with IMA-I needed a re-operation for a postoperative epidural hematoma. 

The three-months postoperative MRI (median = 88.5 days) confirmed large (STR or GTR) resection in 10/30 (33.3%) LG-IMA. One case of IMA-II showed tumoral progression on three-months postoperative MRI despite the surgeon having reported an STR, and one GTR according to the surgical report was converted to STR based on the early postoperative MRI.

### 3.3. Follow-Up

#### 3.3.1. Adjuvant Treatment

Early adjuvant therapy was proposed only in cases where surgery was limited to biopsy. Three patients with IMA-I received CT (two temozolomide, one vinblastine), with adjunction of RT for one of them; two patients with IMA-II were treated by adjuvant RT alone and one patient with IMA-I refused RT (adjuvant therapy being performed at outside institutions, details regarding RT and CT plans were not available).

#### 3.3.2. Recurrences

A radiological tumor progression was observed in 16 patients (53.3% of cases), including 13 cases IMA-I and 3 cases of IMA-II, with a mean delay of 28.5 months after surgery (range = 3–288). 

**Figure 1:**
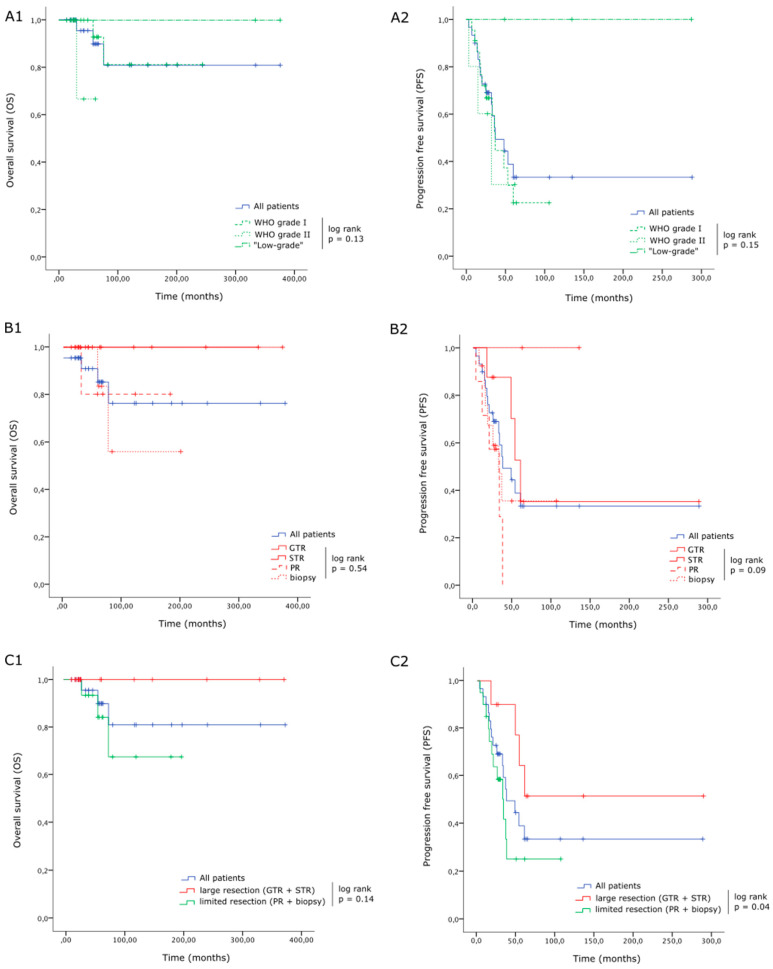
Overall survival (OS) and progression-free survival (PFS) for LG-IMA. There were no significant differences in terms of OS (**A1**) or PFS (**A2**) between WHO grade I and grade II tumors (respectively, 22, 5 and 3 patients with grade I, grade II and “low-grade” IMA). The extent of resection (EOS) was not a significant predictor of OS or PFS when distinguishing each modality individually (GTR, STR, PR and biopsy, including, respectively, 2, 8, 7 and 13 patients; **B1**,**B2**), probably due to the reduced number of patients in each group, but it did make a difference when considering “large resections“ (GTR or STR; 10 patients) versus “limited resections” (PR and biopsies; 20 patients), regarding both OS (**C1**) and PFS (**C2**).

These included: seven biopsies, five PR and four STR. None of the patients with GTR showed tumoral progression (one of the recurring patients had no tumoral remnant visible on the early postoperative MRI but was classified as STR according to the surgical report). Four of the five patients who received an early adjuvant treatment after a biopsy developed recurrence. Twelve out of 16 patients with radiological progression developed symptoms but the other remained asymptomatic until last follow-up (Table 3, Figure 1). 

**Figure 2:**
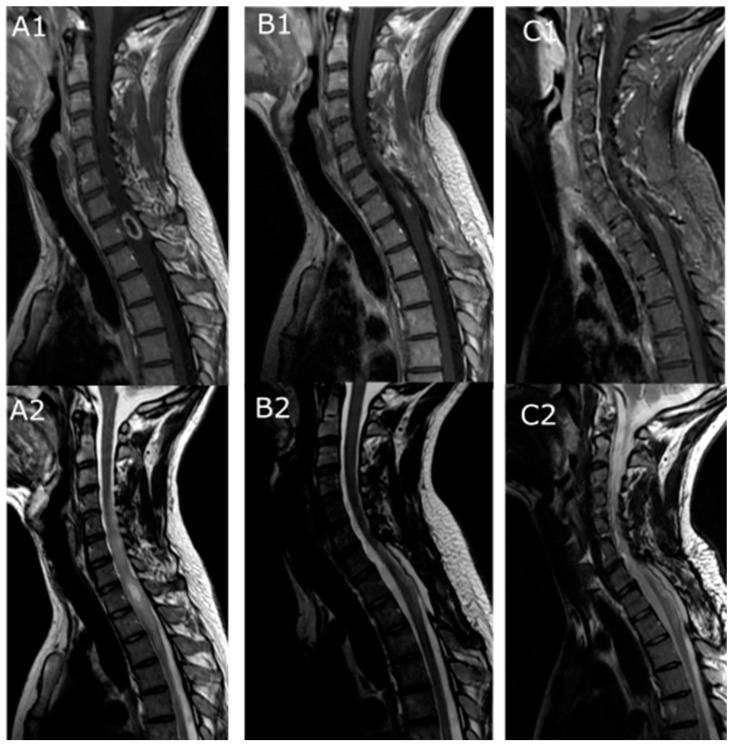
Illustrative cases of recurring LG-IMA. (**A1**,**A2**) Preoperative MRI of a 23-year-old male patient with a T1 intramedullary tumor (upper row: T1-weighted with gadolinium enhancement, lower row: T2-weighted). (**B1**,**B2**) Early (3 months) postoperative MRI after partial resection: histology was in favor of a WHO grade I astrocytoma, (**C1**,**C2**) MRI at 37 months postoperative showing a radiological recurrence. Patient was asymptomatic and was further followed up until 58 months with no symptoms.

Patients that were not symptomatic were managed conservatively (Figure 2). Five patients underwent either revision surgery, followed by CT in one patient and RT with CT in another one. Among the seven patients that were recused for surgery, two had RT alone, two had CT alone, and three underwent RT with CT. At last follow-up, among the 12 patients that underwent retreatment, 6 were stabilized (4 surgery, 1 RT alone and 1 CT alone) while 6 others developed further progression.

#### 3.3.3. Last Follow-Up

Median duration of follow-up was 59 months (range = 13–376). At the last evaluation, only 50% of the patients (63.6% if considering only IMA-I) were able to walk (McCormick grade I and II). Compared to the preoperative status, the individual McCormick grade remained unchanged in 13 patients (43.3%), improved in 2 patient (6.7%) and worsened in 15 patients (50%) (Table 4).

During the follow-up period, five patients presented complications: two patients developed hydrocephalus requiring shunting, one patient developed cervical kyphosis treated by a cervical corpectomy and posterior cervical fusion; one patient developed a meningocele that eventually resolved spontaneously, and one patient treated by temozolomide for tumor recurrence underwent a second surgery for wound dehiscence 3.5 years after initial resection.

Altogether, median overall survival (OS) was 59 months (range = 13–376); OS was 96.3% at 3 years; 90.9% at 5 years and 81.9% at 10 years. Median progression-free survival (PFS) was 28.5 months (range = 3–288); PFS was 51.9% at 3 years; 35.6% at 5 and 10 years. 

There was no significant difference in terms of OS and PFS between WHO grade I and grade II tumors (*p* = 0.13 and *p* = 0.15 respectively, Figure 1. 

The extent of resection (EOS) was not a significant predictor of OS or PFS when distinguishing each modality individually (GTR, STR, PR and biopsy; Figure 1(B1,B2)), probably due to the reduced number of patients in each group, it but did make a difference when considering “large resections“ (GTR or STR) versus “limited resections” (PR and biopsies), regarding both OS (C1) and PFS (C2) (Figure 1C1,C2).

## 4. Discussion

### 4.1. Main Findings

We retrospectively reviewed a single-center series of 40 patients operated on for IMA, including 30 LG-IMAs. We evaluated the long-term outcome after surgery, and particularly the recurrence rate and management for LG-IMA.

Tumor grade is the main prognostic factor for IMA overall [14], with LG-IMA (WHO grade I and II) having a better prognosis in term of OS [4,9,10] than higher grade (WHO grade III and IV). Altogether, OS at 5 and 10 years was 90.9% and 81.9% for LG-IMA. Similarly, Ogunlade et al. [4] reported a rate of OS at 5 years of 82% for grade I, 70% for grade II (versus 28% for grade III and 14% for grade IV) and overall survival at 10 years for LG-IMA reaches 76.8% [11] and 80% [1] according to different authors. However, within low-grade tumors, histological grading (grade I versus grade II) was not significantly associated with survival rates in this study.

In contrast, in our study, the extent of surgical resection was an important prognostic factor even within LG-IMA (for both OS and PFS, even though it did not reach statistical significance for OS, probably due to the low number of patients and mortality events). Remarkably, neither of the two patients with LG-IMA who underwent GTR recurred. It is known that the extent of resection [1,4,7,9,11,15,16] is an important factor for PFS, in addition to histological grading [5,9,10,11,12,13,14]. However, some authors report similar PFS between grade I and grade II IMA [14,16,18] and a prognostic difference was found between grade I and II patients for the same surgery (biopsy versus resection) by Lebrun et al. [14,16,18]. Similarly, Fakhreddine et al. [12] reported no differences in terms of the extent of resection between grades I and II.

Lastly, the first aim of surgery is to prevent neurological worsening. Therefore, the extent of resection must be balanced with the risk of definitive post-operative neurological deficit. We observed neurological worsening of 1 or 2 grades on the McCormick scale in the first evaluation after surgery of 30% in LG-IMA patients and 20% in HG-IMA patients, to 50% and 40% at end of follow-up, respectively. In the literature, the rates of neurological deterioration vary from 18 to 80% [5,25] for IMAs of all grades. Interestingly, extensive resection was not associated with worse post-operative neurological status for IMAs [9], even in LG-IMAs [1]. 

### 4.2. Recurrences

Despite their favorable outcome in term of OS, 53.3% of patients with LG-IMA in our study showed radiological tumor progression after a mean delay of 28.5 months after surgery (range = 3–288). Recurrence was symptomatic in 12/16 patients and occurred in 4/10 patients with either GTR or STR. In comparison, overall recurrence rates of 21.2% [11] and 41.2% [18] were reported in other studies for LG-IMAs. 

The high recurrence rate (53.3%) of LG-IMAs in our series could be explained by the limited extent of resection: a large (GTR or STR) resection could be performed in only 30% of the patients because most of them were infiltrative. Indeed, a limited dissection plan was present in only four LG-IMAs (13.3%); no cleavage plan being found in the other patients. Our results are consistent with those reported by other authors with total resection in IAM varying from 10.7% [7]; 14.3% [18]; 17% [14]; 27.3% [13]; 29.1% [10]; 29.5% [11]; 42.5% [5] to 59.7% [12]. GTR in LG-IMA was associated with better OS, with prevention of neurological worsening [1,26], while PR was associated with worse prognosis and neurological worsening [1]. 

Astrocytoma are infiltrating tumors, a fact which limits the resection and increases recurrence [11]. Even though pilocytic astrocytoma are sometimes described as well-circumscribed tumors [19], in our study, only a limited plane was found in 9.1% of the patients with IMA-1. Lebrun et al. [14] reported no significative differences in terms of infiltrative pattern between IMA-I (67%) and IMA-II (71%) or between rates of total resection in LG and HG-IMA. As a consequence, the absence of cleavage plan during surgery is not sufficient to rule out the diagnosis of IMA-I [19], and Hongo et al. [27] showed that intraoperative frozen-section did not allow a differential diagnosis between intramedullary tumors. Resection should thus not be limited during surgery because of an infiltrating pattern. 

### 4.3. Adjuvant Therapies

Indications for adjuvant treatment remain unclear for LG-IMA. Although several studies recommend postoperative RT after incomplete resection of LG-IMA [3,17,22,23], others did not demonstrate increased PFS or OS after irradiation [1], in particular, without GTR [1], or reported even shorter survival PFS after RT [6,12]. Adjuvant chemotherapy by temozolomide or bevacizumab is also an option [3,17], even if some studies failed to show improvement in survival rates [1,6,12]. Shorter OS/PFS after chemotherapy for IMA-I were also reported [12]. In our study, adjuvant CT and/or RT was considered in LG-IMA only in case of biopsy.

At recurrence, there is also no consensus of treatment for LG-IMA. A reintervention is recommended, with adjuvant treatment by RT or CT in case of residue [11]. Moreover, RT toxicity must be balanced against the risk of symptomatic tumor progression, leading some groups to propose chemotherapy alone for progressive IMA-II [28]. 

In our opinion, recurrences should only be treated when symptomatic. In these young patients with slowly growing tumors, treatment options are limited, and most can only be repeated once (intramedullary surgery or RT). Long-term and well-thought out strategies are therefore paramount.

Only one LG-IMA progressed toward a higher histological grade in our series. In the same way, only 4.3% of IMA underwent transformation in the study of Lebrun et al. [14]. IMA and cerebral astrocytoma behave somewhat differently and the integration of some clinicoradiological criteria with histology for IMA is not helpful as it is for cerebral glioma, LG and HG-IMA both being enhanced by contrast on MRI, for example [14]. 

### 4.4. Molecular Profiling

A recent deeper understanding of the biomolecular characteristics of IMA confirmed that cranial and spinal astrocytomas are different entities [8,14,29], suggesting that IMA may arise from alternative mechanisms of tumorigenesis than their cranial counterparts [5,29]. In contrast to cerebral astrocytoma to which they are classically associated, IDH mutations are rare in IMA, especially for canonic mutations (IDH1 p.R132H and IDH2.R172H) [3,14]. Genomic analysis performed on IMA showed that most frequent mutations seen in the spinal cord are BRAF, the prognostic impact of which is still unclear [8,30,31], and H3K27M [3,14]. LG-IMA, especially IMA-I, are associated with BRAF mutation. Major BRAF mutations are the BRAF V600E mutation [8] and the KIAA1549-BRAF fusion but in the spinal cord, KIAA1549(15)-BRAF(9) fusion is most common, while KIAA1549(16)-BRAF(9) is most common on the brain [14,19]. The H3K27M mutation is the most important mutation in HG-IMA but may not have as poor a prognosis as H3K27M-mutated midline gliomas [8]. The only biomolecular alteration found in IMA-I in our series was a BRAF-mutation, detected in four of six tested IMA-I (66.7%). 

In addition to NGS, methylome analysis will further improve our classification and prognostic stratification for IMA. Multimodal classifiers based on histology, molecular genetics and methylome analysis recently identified new prognostic subgroups for spinal astrocytomas [32].

### 4.5. Limitations

Our present series presented some limitations as a retrospective study of a small cohort of patients related to the rarity of IMA. Our observations need to be confirmed by larger studies. 

Thus, we will include patients operated on at other neurosurgical centers, in France and abroad, in order to lead a larger multicentric study. To integrate the pathological diagnostic criteria with the new molecular data and to evaluate the prognostic value of the several molecular markers, we will perform biomolecular analyses on the tumor samples of those patients. 

## 5. Conclusions

In conclusion, although LG-IMAs are infiltrating tumors, the extent of resection (GTR or STR), but not WHO grading, is the main prognostic factor. The management of recurring tumors is highly variable with no conclusive evidence for either option. Nonetheless, LG-IMAs are slowly progressing tumors with a favorable survival outcome. A more precise definition of molecular alterations in IMA may help to develop targeted therapies and improve individual outcomes. 

## Figures and Tables

**Table 1:** Baseline clinical and radiological features in 30 patients with LG-IMA.

Clinical Features		Number (Total n = 30)
Mean age (years, (min–max))		28.5 (16–66)
Sex ratio (F/M)		18/12
Mean delay between symptoms onset and surgery (months, (min–max))		18 (1–120)
Preoperative McCormick scale	I	5 (16.7%)
	II	19 (63.3%)
	III	5 (16.7%)
	IV	1 (3.3%)
	V	0
Preoperative symptoms	Pain	25 (83.3%)
	Motor	23 (76.7%)
	Sensory	27 (90%)
	Sphincter	13 (43.3%)
Radiological features		
Vertebral levels involved	Mean (min–max)	3 (1–9)
	Cervical	8 (26.7%)
	Cervicodorsal	2 (6.7%)
	Dorsal	18 (60%)
	Dorsolumbar	2 (6.7%)
	Cone	0
	Panmedullary	0
T1-weighted sequence signal (%)	Hypo	11/23(47.8%)
	Iso	10/23 (43.5%)
	Hyper	2/23 (8.7%)
T2-weighted sequence signal (%)	Hypo	2/24 (8.3%)
	Iso	0
	Hyper	22/24 (91.7%)
Gadolinium enhancement		26 (86.7%)
Peritumoral cyst		18/29 (62%)
Peritumoral hemorrhage		9/28 (32.1%)

**Table 2:** Surgical and early postoperative results in 30 patients with LG-IMA.

Surgery		Number (Total n = 30)
Cleavage plan described		4 (13.3%)
Residue according to surgeon		28 (93.3%)
Residue visible on first post-op MRI		27 (90%)
Extent of resection according to surgeon	Gross total resection	2 (6.7%)
	Subtotal resection	8 (26.7%)
	Partial resection	7 (23.3%)
	Biopsy	13 (43.3%)
Pathology		
Ki67 levels	<5%	20/22 (90.9%)
	≥5%	2/22 (9.1%)
Clinical		
Early postoperative McCormick scale	I	5 (16.7%)
	II	13 (43.3%)
	III	7 (23.3%)
	IV	4 (13.3%)
	V	1 (3.3%)
Complications	Epidural hematoma	1 (3.3%)

**Table 3:** Detailed history of recurrence progressions in 16 patients with LG-IMA.

Sex	Age	Spinal Segment	PreopMCS	Extent of Resection	WHO Grade	Ki-67 (%)	Postop MCS	Early Adjuvant Treatment	Delay for Progression (Months)	Management of Progression
M	37	Dorsal	III	Biopsy	I	5	IV	No	7	Surgery
F	33	Cervical	I	Biopsy	I	10	I	No	36	Surgery + RT + CT
F	22	Cervical	II	PR	I	3	III	No	53	RT
F	44	Cervical	II	PR	I	1	I	No	33	CT
F	47	Cervico-dorsal	II	PR	I	1	III	No	11	RT + CT
F	24	Dorsal	II	STR	I	1	II	No	48	Surgery
M	23	Dorsal	II	PR	I	4	II	No	37	No treatment
F	49	Dorso-lumbar	II	Biopsy	I	2	III	No	25	RT + CT
M	30	Dorsal	II	Biopsy	I	-	IV	CT	18	RT + CT
F	44	Cervical	II	STR	I	3	III	No	60	No treatment
F	16	Dorsal	II	Biopsy	I	5	II	CT	14	Surgery + CT
F	37	Dorsal	II	STR	I	-	II	No	17	No treatment
F	40	Dorsal	II	PR	I	2	II	No	20	Surgery
M	27	Cervical	III	STR	II	10	III	No	3	RT
M	58	Dorsal	II	Biopsy	II	1	II	RT	15	No treatment
F	16	Dorsal	IV	Biopsy	II	6	IV	RT	32	CT

**Table 4:** Clinical and radiological outcome at last follow-up.

		Number (Total n = 30)
Follow-up	Mean (in months)	90.3
	Median (min–max)	59 (13–376)
McCormick scale at last follow-up	I	3 (10%)
	II	12 (40%)
	III	8 (26.7%)
	IV	4 (13.3%)
	V	3 (10%)
Tumor progression	PFS (in months)	28.5 (3–288)
	PFS (in years)	2 (0–24)
	Radiological	16 (53.3%)
	Symptomatic	12 (40%)

## Data Availability

Clinical and radiological data are available upon reasonable request for the corresponding author.
